# Smokeless Tobacco and Oral Cancer in South Asia: A Systematic Review with Meta-Analysis

**DOI:** 10.1155/2014/394696

**Published:** 2014-07-06

**Authors:** Zohaib Khan, Justus Tönnies, Steffen Müller

**Affiliations:** ^1^Leibniz Institute for Prevention Research and Epidemiology-BIPS, Achterstraße 30, 28359 Bremen, Germany; ^2^Khyber Medical University, Peshawar 25000, Pakistan

## Abstract

*Introduction*. Smokeless tobacco is considered one of the major risk factors for oral cancer. It is estimated that over 90% of the global smokeless tobacco use burden is in South Asia. This paper aims to systematically review publications reporting epidemiological observational studies published in South Asia from 1984 till 2013. *Methods*. An electronic search in “Medline” and “ISI Web of Knowledge” yielded 734 publications out of which 21 were included in this review. All publications were assessed for quality using a standard quality assessment tool. Effect estimates (odds ratios (OR)) were abstracted or calculated from the given data. A random effects meta-analysis was performed to assess the risk of oral cancer with the use of different forms of smokeless tobacco. *Results and Conclusion*. The pooled OR for chewing tobacco and risk of oral cancer was 4.7 [3.1–7.1] and for paan with tobacco and risk of oral cancer was 7.1 [4.5–11.1]. The findings of this study suggest a strong causal link between oral cancer and various forms of smokeless tobacco. Public health policies in affected countries should consider SLT specific cessation programs in addition to campaigns and activities incorporated into smoking cessation programs.

## 1. Introduction

Oral cancer is one of the most common noncommunicable diseases worldwide with an estimated increase of 275,000 new cases each year [1]. Oral cancer is the term used for cancers that form in tissues of the oral cavity (the mouth) or the oropharynx (the part of the throat at the back of the mouth) [2]. These along with other head and neck cancers are the sixth most prevalent type of cancer in the world [3, 4] and one of the leading causes of death in developing countries [5]. The countries of South Asian region including India, Pakistan, Afghanistan, Bangladesh, Sri Lanka, Bhutan, Nepal, Iran, and Maldives [6] are particularly affected, with oral cancer ranking either first or second with regard to different types of cancer prevalence in these countries [7].

The reasons for the high prevalence of head and neck cancers in South Asia have been investigated to some extent but, as is the case with most developing countries, a lack of research infrastructure has put constraints on studying the epidemiology of these conditions in the context of South Asia [8]. One of the major risk factors associated with the high prevalence of head and neck cancer and oral potentially malignant diseases (OPMD) in this region is smokeless tobacco (SLT) [9]. It is estimated that over 90% of the global smokeless tobacco use burden is in South East Asia [10]; around 100 million people use smokeless tobacco in India and Pakistan alone [11]. SLT is used in many forms varying from chewing tobacco not mixed with any other ingredient to a mixture of tobacco with other ingredients such as in betel quid, areca nut with tobacco, Naswar, paan-masala with tobacco, Gutkha, Khaini, and Mishri [12, 13]. Smokeless tobacco contains around 28 known carcinogens. These include the nonvolatile alkaloid-derived tobacco-specific N-nitrosamine and N-nitrosamino acids as the major group while volatile tobacco-specific nitrosamines, volatile aldehydes, and some poly nuclear agents have also been shown to be present in smokeless tobacco [14].

With such a high prevalence of both SLT use and oral cancer in the South Asian region, it is of utmost importance that epidemiological research is carried out to carefully assess their detailed relationship. Two published reviews coauthored by IARC researchers have focused on overall associations found in studies worldwide [15, 16]. Several overviews originating from South Asia have been published on oral cancer and smokeless tobacco [15, 17–26] but to date no systematic review of the published literature on association of oral cancer with different forms of smokeless tobacco focusing specifically on South Asia has been conducted. This paper aims to address the issue by systematically reviewing publications reporting epidemiological observational studies carried out/published in the South Asian region during the last 30 years, that is, published after 1984 on the use of all forms of SLT and its relationship with oral cancer.

## 2. Methods

### 2.1. Search Strategy

An electronic search was carried out in “Medline” and “ISI Web of Knowledge” in August 2013. Various combinations of the terms “oral premalignant disease,” “oral precancer,” “leukoplakia,” “erythroplakia,” “submucous fibrosis,” “lichen planus,” “oral cancer,” “oral carcinoma,” “mouth neoplasm,” “head and neck cancer,” “squamous cell carcinoma of the oral cavity,” “carcinoma lip,” “carcinoma tongue,” “oral neoplasms,” and “head and neck neoplasms”; “smokeless tobacco,” “Naswar,” “paan,” “snuff,” “oral snuff,” “chewing tobacco,” “betel quid,” “areca nut,” and “Gutkha”; “Pakistan,” “India,” “Bangladesh,” “Iran,” “Sri Lanka,” “Afghanistan,” “Bhutan,” “Nepal,” “Maldives” were used. The terms for different OPMDs were included in the search process as sometimes these conditions are studied together with oral cancer. No filters were used during the electronic searches.

### 2.2. Publication Selection

The following selection criteria were applied to all the publications returned by the electronic searches to be included in the review.

#### 2.2.1. Inclusion Criteria

Inclusion criteria are as follows:

- papers published after 1984,
- epidemiological observational study in humans of cohort or case-control design,
- studies carried out in “South Asia” according to the United Nations geographical region classification (including the following countries: India, Pakistan, Afghanistan, Bangladesh, Sri Lanka, Bhutan, Nepal, Iran, and Maldives),
- reported outcome or one of the reported outcomes is oral cancer or head and neck cancer, and
- exposure to paan, Gutkha, betel nut, areca nut, or any other type of smokeless tobacco.

#### 2.2.2. Exclusion Criteria

Exclusion criteria are as follows.

- Studies reporting oesophageal, base of the tongue, and salivary glands cancers were excluded.
- Studies involving laboratory research and molecular/genetic epidemiology were excluded.

The selection process was done in three steps: first, the titles of all publications were scanned and relevant publications selected. The next step involved reading the abstracts of the publications selected in the first step. Full text of the publications identified during step two were then obtained. The selected publications were then divided into three groups according to their reported outcomes: (1) OPMD as an outcome, (2) oral cancer as an outcome, and (3) OPMD and oral cancer as an outcome; publications reporting only OPMDs as an outcome were excluded at this stage. Reference lists of the selected publications were scanned to identify any additionally relevant publications.

### 2.3. Quality Assessment

All selected publications were assessed for their quality on the basis of the “Effective Public Health Practice Project Quality Assessment Tool for Quantitative Studies” [27]. Studies were ranked as “strong,” “moderate,” and “weak” after being assessed on six parameters, that is, selection bias, study design, confounding, blinding, data collection methods, and withdrawals and dropouts. Quality assessment was carried out by two authors independently and the results were later compared. Any differences were discussed in the presence of all three authors and a final decision was reached by mutual consensus.

### 2.4. Data Extraction

Data extraction was carried out between October and December 2013. First, data regarding the study type, location of the conducted study, sample size, year of publication, exposure, outcome, and the effect size were tabulated separately by two authors and later compared in the presence of all three authors. The data were then divided into two broad groups according to the difference in the type of SLT exposure, that is, “paan or betel quid with tobacco” and “chewable tobacco which included all types of smokeless tobacco other than paan.” Adjusted odds ratios (OR) along with their 95% confidence intervals (95% CI), if reported, were recorded. In studies, where OR were not reported but the data required to calculate them were available, OR were calculated using a Mantel-Haenszel (MH) approach, thus providing us with weighted OR across the different strata reported. However, if the paper did not report an adjusted OR and the data given were too scarce to calculate MH-OR, then the crude OR as reported in the paper or calculated from the given data was recorded. OR were also recorded or calculated for male and females separately, total duration of the habit in years, and frequency of daily use. Again efforts were made to record the most adjusted measure, whenever permissible. Standard errors of the natural logs of the OR were calculated either from the 95% CI of the respective log OR or by using the formula $\text{SE}(\ln{\text{OR}}_{\text{MH}})=\sqrt{\sum (b_{i}c_{i}/N_{i})²v_{i}/\left(\sum b_{i}c_{i}/N_{i}\right)²}$ when a MH-OR was calculated.

### 2.5. Meta- and Heterogeneity Analysis

During the data extraction stage it had become obvious that there was major heterogeneity regarding methodological and other parameters among the selected publications. Nevertheless all data were entered into Rev Man 5.2 [28] and meta-analyses performed across all exposure categories, and their effect on oral cancer separately and combined was recorded. This was done with the inverse variance method using both fixed and random effects. This also provided the *I* ^2^ estimates of statistical heterogeneity. The *I* ^2^ estimate was used to assess heterogeneity as it provides a better estimate for quantifying heterogeneity. Heterogeneity was considered low if the *I* ^2^ estimate was below 25%, moderate if it was between 25 and 50%, high if it was between 50 and 75%, and very high above the value of 75%. Due to a very high level of heterogeneity, random effect meta-analysis has been used for this review. Sensitivity and influence analysis were done by excluding one study at a time and checking its effect on the pooled estimate and the heterogeneity, but this had little effect on lowering the *I* ^2^ statistic.

Meta-analyses were performed for overall estimates, case-control studies, studies with hospital controls, cohort studies, studies from India only, studies from southern India only, studies for Maharashtra state, studies adjusting for smoking and/or alcohol, studies with moderate quality, and studies involving only men.

### 2.6. Narrative Synthesis

For the categories where the data was incomplete, unavailable, or calculated using different methods, for example, the exposure response categories, a narrative synthesis was done. The synthesis highlights the highest and lowest estimates in general, according to gender and for studies that had done adjustment for alcohol and/or smoking.

## 3. Results

**Figure 1:**
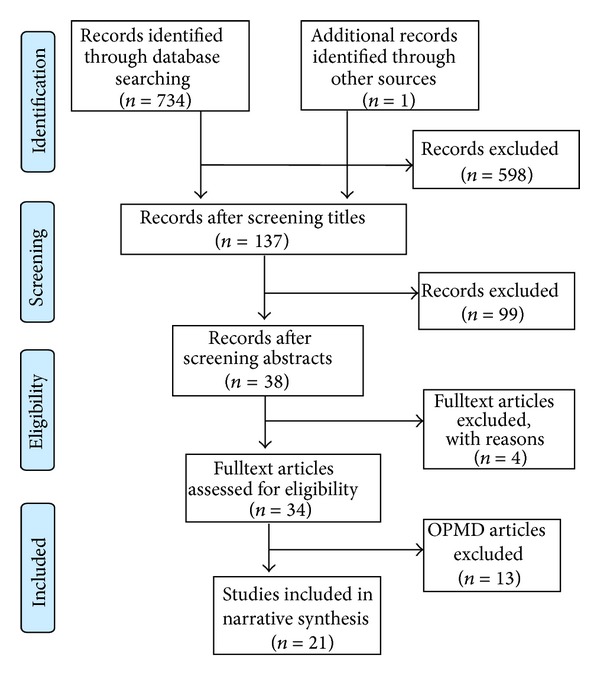
Flow chart of selection process of articles included in the review.

A total of 734 publications were identified from both database searches (Medline, ISI Web of Knowledge) (Figure 1). One more paper was identified from a supplementary web search but it just reported the findings from one of the included studies and hence was excluded. After the first round of exclusion 137 publications remained; after reading the abstracts, 38 publications were selected and their full text versions obtained. 4 publications were excluded after examining the full paper. This left us with a total of 34 publications. 21 publications reported oral cancer as the outcome or one of the outcomes and 13 publications reported just OPMD as the outcome. The publications corresponding to OPMD were excluded at this stage.

The 21 publications [29–49] for oral cancer included in this review correspond to 19 different studies and three studies were of cohort design while the remaining were of case-control design (Table 1). Two studies were carried out in Pakistan and the rest in India. 13 publications were published in or after the year 2000 while the remaining publications were published before the year 2000, the oldest publication being from 1989.

11 of the selected publications reported or contained data on paan with tobacco (betel quid) as a risk factor whereas 14 publications reported or contained data on chewing tobacco other than paan or without specifying any particular type of SLT. 11 publications reported or contained data stratified by sex.

Data regarding daily frequencies of smokeless tobacco use were reported in 14 publications, while data on the total duration of the habit was reported in 10 publications. Table 1 includes all selected studies for oral cancer and their features along with the quality assessment result for each study.

The values for *I* ^2^ statistic ranged from 77% to 96% when pooling studies across different strata. Core findings from the included publications are given in Table 1. Additional characteristics of the included studies are presented in the supplementary Table  1 available online at http://dx.doi.org/10.1155/2014/394696.

For the purpose of clarity and taking into consideration the considerable difference between the outcome estimates related with the use of betel quid and other forms of SLT, we reviewed the relationship of oral cancer with SLT in two groups: (1) chewing tobacco of all kinds excluding betel quid or paan with tobacco and (2) betel quid or paan with tobacco.

### 3.1. Chewing Tobacco and Oral Cancer

**Figure 2:**
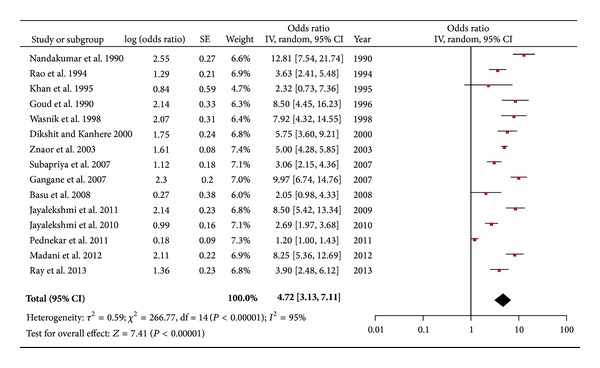
Forest plot of chewing tobacco and risk of oral cancer.

Overall 14 publications reported different forms of chewing tobacco, predominantly Gutkha and chewing tobacco leafs (Table 2). Five publications reported OR that had been adjusted for smoking among other confounders. The adjusted OR ranged from 3.6 [2.5–5.6] [34] to 8.3 [5.4–13] [48]. The OR ranged from 1.2 [1.0–1.4] [47] to 12.9 [7.5–22.3] [33] among the publications in which either crude odds ratios were mentioned or a MH-OR was calculated from the given data. The pooled OR for chewing tobacco and risk of oral cancer was 4.7 [3.1–7.1] (Figure 2). The studies where adjustment for alcohol and/or smoking had been done, when combined, provided a pooled OR of 4.3 [3.1–5.8]. The pooled OR from combining only case-control studies was 5.4 [4.1–7.1]. Case-control studies having hospitals as a source of controls when combined gave a pooled estimate of 4.2 [2.5–6.9]. Cohort studies when combined provided a pooled OR of 2.9 [1.1–8.3]. For studies carried out in India the pooled estimate was 4.8 [3.2–7.4]. For studies carried out in South India, which comprises of the states of Andhra Pradesh, Kerala, Karnataka, and Tamil Nadu, the pooled OR was 5.1 [3.3–8.1]. The pooled OR for studies carried out in the state of Maharashtra was 4.8 [1.7–13.5]. When studies of moderate quality were combined, the pooled estimate came out to be 4.5 [2.8−7.3]. The pooled estimate for studies ranked as “weak” was 5.2 [2.6–10.3].

#### 3.1.1. Gender Differences

Three publications reported or contained data from which OR for men and/or women could be calculated separately (Table 2). Among men the OR ranged from 1.2 [1.0–1.4] [47] to 5.8 [3.6–9.5] [37]. Only two studies reported OR separately for women ranging from 6.4 [3.3–9.0] [49] to 25.3 [11.2–57.3] [33]. Studies carried out with only men taken as study subjects when combined provided a pooled OR of 4.0 [2.9–5.7].

#### 3.1.2. Exposure-Response Relationships

*Intensity/Frequency.* A total of seven publications provided dose response relationships according to the intensity of daily usage as exposure metric (Table 2). These OR varied from 1.1 [1.0–1.4] [47] for chewing tobacco or chewable products containing tobacco for less than 5 times a day to 20.0 [8.1–48.9] [36] for more than 10 times a day compared to nonchewers; among studies adjusted for smoking and/or alcohol the corresponding values were 2.0 [1–3.8] and 13.9 [7.1–27.2], both coming from the same study done by Diskshit et al. [37].

*Duration of Use.* Six publications described the effect of chewing tobacco on developing oral cancer in terms of the total duration of the habit (Table 2). The OR varied from 0.8 [0.4–1.7] [47] for the total duration of the habit being less than 10 years, compared to nonchewers, to 10.9 [5.9–20.0] [36] for a usage duration of 20 years or more compared to nonchewers.

### 3.2. Paan/Betel Quid (with Tobacco) and Oral Cancer

**Figure 3:**
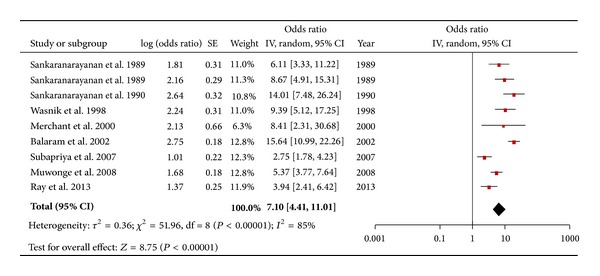
Forest plot of betel quid plus tobacco and the risk of oral cancer.

A total of nine publications included in this review reported OR or contained data from which OR could be calculated for the risk of chewing paan/betel quid and oral cancer (Table 3). Six publications [29–31, 38, 41, 44] reported overall OR which were adjusted for confounding factors such as smoking and/or alcohol. The adjusted OR varied from 3.1 [41] to 14.1 [7.4–26.5] [31]. Overall, the OR (both adjusted and unadjusted) varied from 3.1 [41] to 15.7 [11.0–22.1] [39]. The pooled OR for chewing paan/betel quid and risk of oral cancer was 7.1 [4.5–11.1] (Figure 3). The studies where adjustment for alcohol and/or smoking had been done, when combined, provided a pooled OR of 6.3 [3.9–10.2]. Case-control studies having hospitals as a source of controls when combined gave a pooled estimate of 7.4 [4.4–12.4]. For studies carried out in India the pooled estimate was 7.0 [4.4–11.1]. For studies carried out in South India the pooled OR was 7.4 [4.1–13.0]. Only one study was carried out in the state of Maharashtra where the OR was 9.3 [5.1–17.2]. When the one “weak” study, for which the OR was 3.9 [2.4–6.4], was excluded, the pooled estimate came out to be 7.6 [4.7–12.3]. Similarly the pooled risk estimates from studies carried out in South India were comparatively higher than the overall pooled estimate.

#### 3.2.1. Gender Differences

Six studies reported or contained data from which OR could be calculated separately from men and/or women (Table 3). For men the OR for chewing betel quid with tobacco ranged from 1.5 [0.75–3.02] [49] to 10.9 [31]; among women the OR ranged between 6.5 [29] and 45.8 [25−84.1] [39].

#### 3.2.2. Exposure-Response Relationships

*Intensity/Frequency.* Five studies reported the effect of frequency of daily use of paan with tobacco on oral cancer (Table 3). The OR varied from 3.3 [1.6–6.9] [29] for chewing paan with tobacco, for less than 5 times a day compared to nonchewers, to 24.7 [12.5–48.7] [39] for someone chewing it more than 10 times a day compared to nonchewers; for studies adjusted for smoking and/or alcohol the corresponding values were 3.3 [1.6–6.9] [29] and 15.7 [31].

*Duration of Usage.* Four studies reported OR for the total duration of habit and oral cancer (Table 3, last column). The OR for chewing habit duration varied from 3.4 [30] for a chewing habit of less than 10 years to 14.6 [30] for a chewing habit persisting for 20 years or more; the corresponding values for studies adjusting for smoking and/or alcohol were 3.4 and 14.6 both from the same study by Sankarnarayanan et al. [30].

## 4. Discussion

The results of this systematic review suggest a strong link between different forms of smokeless tobacco (SLT) and oral cancer and further strengthens and supports the IARC's take on SLT that it is a risk factor for oral cancer [15, 16]. Users of betel quid with tobacco have a sevenfold higher risk for developing oral cancer as compared to nonchewers, OR 7.1 [4.5–11.1]. This finding is consistent with findings from the earlier reviews [15, 16]. Similarly, people using other forms of SLT than betel quid with tobacco have an almost five-time higher risk of developing oral cancer as compared to nonchewers, OR 4.7 [3.1–7.1]. These increased risks were consistently significant even after adjustment for other risks factors such as alcohol and smoking; that is, pooled OR for betel quid with tobacco after adjustment for alcohol and smoking was 6.3 [3.9–10.2] and the corresponding value for the chewing tobacco group was 4.3 [3.1–5.8]. These pooled estimates, however, should be dealt with caution because of the high levels of heterogeneity present among the studies but, despite indications of heterogeneity, even the lowest effect estimates among the individual studies are above the value of 1, pointing towards a causal link between SLT and oral cancer. The large variability of the effect estimates among individual studies may be attributed to differences in the composition of the products and population characteristics across the region. Additionally, although most studies are case-control design, there are differences between the sources and ratio of controls to the number of cases. In general, the three cohort studies provide relatively conservative estimates as compared to the case-control studies (Table 1).

For the chewing tobacco category, case-control studies provided a pooled estimate, OR 5.46 [4.1–7.1], which was significantly higher than that of the cohort studies, OR 2.9 [1.0–8.3]; albeit the pooled estimate for the cohort studies had an increased width of the confidence intervals. This finding is in contrast with the review done by Guha et al. [15], where they reported a higher pooled estimate for cohort studies as compared to the case-control ones. This may be explained by a difference in the selected cohort studies, as this review has only one cohort study in common with that review. They have included two cohort studies published prior to 1983, which might have reported considerably higher risk estimates. The source of controls had only a slight bearing on the pooled estimates, with the pooled OR for combining studies where controls were taken from hospitals, being slightly lower as compared to studies where population controls were recruited. This is consistent with previous findings [15]. The pooled OR for studies carried out in South India and the state of Maharashtra are relatively higher than the overall pooled estimate and this might be explained by the relatively high prevalence of SLT use in these geographic locations [9, 13, 50] and incidence of oral cancer [51]. The quality of the combined studies had minimal effect on the overall summary estimate, that is, OR 4.5 [2.8–7.3], compared to the overall pooled OR of 4.7 [3.1–7.1]; however, in the chewing tobacco group exclusion of the weak studies (*n* = 4) lowered the pooled estimate, while in the betel quid group, where there was just one weak study, the overall estimate increased when the “weak” study was excluded. The studies which were ranked as “weak” did not play any role in the narrative synthesis either; most had not reported any results or suitable data for calculation of ORs, in the exposure response categories of frequency/intensity and duration.

Paan with tobacco appears to have a higher risk as compared to chewing other tobacco products; the overall pooled OR for paan as well as the pooled OR across different exposure strata are significantly higher in comparison with the other forms of chewing tobacco (Figure 3 and Tables 2 and 3). A possible reason for this could be the use of areca nut in paan, as it has been shown to have carcinogenic properties on its own [52] and thus might have a synergistic effect with the carcinogenicity of SLT, resulting in a higher risk of oral cancer as compared to other forms of SLT use. Similarly another ingredient, slaked lime, used in betel quid preparation has been shown to have carcinogenic potential. It facilitates the production of reactive oxygen species (ROS) in the saliva of chewers and also facilitates the hydrolysis of arecoline into arecaidine which in turn facilitates increased fibroblast proliferation and collagen synthesis, which are essential for premalignant and malignant transformation of the affected tissues [53]. The betel quid with tobacco group analysis included only case-control studies and therefore a formal comparison of the risk estimates among case-control and cohort studies could not be done. However, similar to the chewing tobacco group, the studies which recruited hospital controls had a relatively higher pooled risk estimate, that is, OR 7.4 [4.4–12.2], compared to the overall estimate.

An interesting observation is the risk differences among males and females, with females being at a significantly higher risk of oral cancer from SLT use as compared to men (Tables 2 and 3). This may be attributed to increased susceptibility of the female oral mucosa to damage by tobacco products [39] and relative lack of education and poverty, all of which have been shown to be significant risk factors on their own [9, 22]. Also it may be due to a lower background risk for oral cancer among women of this region because of a lower prevalence of smoking and alcohol drinking [15]. Also a high prevalence of cervical cancer among women in India [54] may be suggestive of the presence of human papilloma virus (HPV) [55], which is an established risk factor for oral cancer as well. There is, however, significant inconsistency in effect estimates among the case-control studies regarding risks in women and also between the case-control and the cohort studies, which might have led to an overestimation of the risk estimates among women. In the study carried out by Jayalekshmi et al. where cohorts of men and women were analyzed separately [45, 46], the authors found that the risk estimates were almost similar among both sexes, which underscores the argument that the true effect size for the relationship between SLT and oral cancer in women may be overestimated. However, it should be clear that, regardless of the magnitude of effect size, all included studies that provide sex-specific estimates provide evidence that SLT is a major risk factor for oral cancer among women in the South Asian region. These results may warrant future research to specifically focus on sex differences and provide reliable risk estimates among men and women using SLT.

The results of our review suggest that there is an exposure-response causal relationship between SLT use and oral cancer, for both the intensity and duration of use. This effect is somewhat linear in case of the chewing tobacco group but for the betel quid group, though the data suggests a possible relationship, it is a nonlinear one. This result is consistent with the IARC reviews but differs from findings of some other reviews [56–58] carried out on published literature from North America and Europe. In these reviews no dose response relationships were identified. This may be explained by the difference in the types of SLT used in South Asia compared to North America and Europe. Differences in ethnicity and socioeconomic status and environmental differences may be additional reasons for these conflicting findings. It may also be noted that the effect sizes reported in the reviews of studies carried out on SLT and oral cancer in Europe and North America report significantly smaller observed effect estimates as compared to the studies included in the present review. The synthesis of the reviewed publications suggests that the total duration of exposure to SLT increases the risk of oral cancer; that is, subjects who used SLT (chewing tobacco, paan/betel quid) for more than 10 years were at a higher risk of oral cancer than those who used SLT for less than 10 years. Parallels can be drawn here with the habit of smoking and alcohol use, where the risk for developing oral cancer increases with an increase in the total duration of exposure to these substances [59]. Furthermore, the mean age of oral cancer cases in the included studies was mostly in the fifth decade of life (Table 1). Given that usually habits like SLT use are generally taken up in early adolescence, this might suggest that prolonged exposure to SLT increases the risk of oral cancer, although age itself has been shown to be a risk factor for oral cancer.

## 5. Limitations of the Review

Some of the limitations are inherent to the observational study designs included in this systematic review, such as recall and selection bias, under-/overreporting of exposure status, retrospective exposure assessment, and uncontrolled confounding. Our electronic search included terms for all countries comprised in the South Asian region, but only publications from India and Pakistan were included because no case-control or cohort studies could be found for other countries. Therefore the results may not be applicable to the entire region. Due to a lack of resources a metaregression analysis could not be performed to identify the sources of heterogeneity; however, in the most recent review done by IARC researchers [15], which includes most of the studies included in our review, metaregression analysis did not lower heterogeneity to moderate or low levels.

## 6. Policy Implications

Given the various types of SLT used in the Indian subcontinent and its increasing popularity in the neighboring countries [60], it is of great importance that the general public be made aware of SLT use as a major risk factor for oral cancer. Most of the tobacco control initiatives around the world have been aimed towards cessation of smoking, where the main strategy to decrease smoking prevalence is the high amount of taxes levied on smoking products. Although this might be productive for smoking cessation, this strategy may facilitate an unintentional push towards smokeless tobacco use and increasing prevalence because SLT is cheaper compared to smoking. Additionally, big tobacco companies revert to manufacturing smokeless tobacco products and advertising them as less harmful than smoking [61]. All these scenarios may potentially lead to a surge in the use of smokeless tobacco products and subsequent increased risks for oral cancer for the general public. The governments and general public should be made aware of the potential dangers related to such approaches and may consider new programs for smokeless tobacco cessation or incorporate the risks of SLT consumption into smoking cessation programs.

## 7. Conclusion

From the published literature it appears that various forms of smokeless tobacco used in South Asia should be considered as strong risk factors for oral cancer. Public health policies in affected countries should consider SLT cessation programs in addition to campaigns and activities to inform the general public about SLT use and oral cancer risks.

## Supplementary Material

The supplementary table: provides additional details about the characteristics of the included publications such as the site of cancer, name of the city/s where the study was conducted, type of controls used (hospital or population), sexes involved in the study and length of the followup for cohort studies etc.

## Figures and Tables

**Table 1:** Characteristics of included studies on oral cancer.

Authors	Year	Location	Study type	Sample Size (cases/controls) (Cohort size/oral cancer cases)∗∗	Quality assessment∗	Mean age of cases	Adjustment for smoking and alcohol
Sankaranarayanan et al. [29]	1989	India	Case-control	228/453	Moderate	n/a	Smoking and alcohol
Sankaranarayanan et al. [30]	1989	India	Case-control	187/895	Moderate	n/a	Smoking and alcohol
Goud et al. [32]	1990	India	Case-control	102/102	Weak	53	No
Nandakumar et al. [33]	1990	India	Case-control	348/348	Moderate	54.8	No
Sankaranarayanan et al. [31]	1990	India	Case-control	414/895	Moderate	n/a	Smoking and alcohol
Rao et al. [34]	1994	India	Case-control	713/635	Moderate	50.35	Smoking and alcohol
Khan et al. [35]	1995	Pakistan	Case-control	24/24	Moderate	54	No
Wasnik et al. [36]	1998	India	Case-control	123/246	Moderate	n/a	No
Dikshit and Kanhere [37]	2000	India	Case-control	558/260	Moderate	n/a	Smoking
Merchant et al. [38]	2000	Pakistan	Case-control	79/149	Moderate	49	Smoking and alcohol
Balaram et al. [39]	2002	India	Case-control	591/582	Moderate	n/a	No
Znaor et al. [40]	2003	India	Case-control	1563/3638	Moderate	n/a	Smoking and alcohol
Subapriya et al. [41]	2007	India	Case-control	388/388	Moderate	50.85	No
Gangane et al. [42]	2007	India	Case-control	140/380	Weak	n/a	No
Basu et al. [43]	2008	India	Case-control	110/110	Weak	n/a	No
Muwonge et al. [44]	2008	India	Case-control	282/1410	Moderate	n/a	Smoking and alcohol
Jayalekshmi et al. [45]	2009	India	Cohort study	79593/92∗∗	Moderate	n/a	No
Jayalekshmi et al. [46]	2011	India	Cohort study	66277/160∗∗	Moderate	n/a	No
Pednekar et al. [47]	2011	India	Cohort study	87222/1267∗∗	Moderate	n/a	No
Madani et al. [48]	2012	India	Case-control	350/350	Moderate	n/a	Smoking and alcohol
Ray et al. [49]	2013	India	Case-control	698/948	Weak	n/a	No

∗Based on the “Effective Public Health Project Quality Assessment Tool for Quantitative Studies”.

∗∗Size of the cohort and the number of oral cancer cases in the cohort.

**Table 2:** Epidemiological studies of chewing tobacco and oral cancer.

Authors	OR (95% CI)	Men OR (95% CI)	Women OR (95% CI)	Frequency/day∗∗			Duration of use in years∗∗∗		
				Tobacco ≤ 5 OR (95% CI)	Tobacco 6–10 OR (95% CI)	Tobacco > 10/OR (95% CI)	Tobacco ≤ 10 yrs/OR (95% CI)	Tobacco 11–20 yrs/OR (95% CI)	Tobacco > 20 yrs/OR (95% CI)
Goud et al. [32]	8.5 (4.3–16.5)	n/a	n/a	8.2 (3.0–22.3)	4.7 (2.0–10.7)	18.4∗	n/a	4.2∗	10.2∗
Nandakumar et al. [33]	12.9 (7.5–22.3)	3.6 (1.7–7.9)	25.3 (11.2–57.3)	9.3 (4.9–17.5)	12.8 (6.6–25.0)	16.6 (6.3–44.3)	n/a	n/a	n/a
Rao et al. [34]	3.6 (2.5–5.6)	3.6 (2.5–5.6)	n/a	n/a	2.8 (2.2–3.5)^+^	3.8∗	1.2 (0.9–1.8)	3.9 (2.7–5.7)	4.1∗
Khan et al. [35]	2.3 (0.7–7.4)	n/a	n/a	n/a	n/a	n/a	n/a	n/a	n/a
Wasnik et al. [36]	7.9 (4.1–13.5)	n/a	n/a	2.1∗	8.1 (3.7–17.9)	20.0 (8.1–48.9)	n/a	n/a	10.9 (5.9–20.0)
Dikshit and kanhere [37]	5.8 (3.6–9.5)	5.8 (3.6–9.5)	n/a	2.0 (1.0–3.8)	6.7 (3.7–12.1)	13.9 (7.1–27.2)	n/a	n/a	n/a
Znaor et al. [40]	5.0 (4.2–5.9)	5.0 (4.2–5.9)	n/a	5.0∗	11.9 (8.9–15.9)^++^	n/a	n/a	3.1 (2.5–3.8)^+++^	9.5∗
Gangane et al. [42]	10.0 (6.7–14.8)	n/a	n/a	n/a	n/a	n/a	n/a	n/a	n/a
Subapriya et al. [41]	2.9∗	n/a	n/a	n/a	n/a	n/a	2.9∗	2.5∗	2.7∗
Basu et al. [43]	2.0 (0.9–4.4)	n/a	n/a	n/a	n/a	n/a	n/a	n/a	n/a
Jayalekshmi et al. [45]	5.5 (3.3–9.0)	n/a	5.5 (3.3–9.0)	3.3 (1.7–6.4)	7.8 (4.4–13.9)	9.2 (4.5–18.7)	n/a	n/a	n/a
Jayalekshmi et al. [46]	5.4 (3.0–9.0)	5.4 (3.0–9.0)	n/a	1.9 (1.2–2.8)	n/a	n/a	n/a	n/a	n/a
Pednekar et al. [47]	1.4 (1.0–2.1)∗∗∗∗	1.4 (1.0–2.1)	n/a	1.1 (0.9–1.4)	1.1 (0.9–1.4)	n/a	0.8 (0.4–1.7)	1.0 (0.7–1.4)	1.1 (1–1.4)
Madani et al. [48]	8.3 (5.4–13.0)	n/a	n/a	n/a	n/a	n/a	n/a	n/a	n/a
Ray et al. [49]	3.9 (2.4–6.1)	2.8 (1.5–5.1)	6.4 (3.2–12.7)	n/a	n/a	n/a	n/a	n/a	n/a

OR: odds ratio, CI: confidence interval, ∗95% CI not reported and/or could not be calculated, ∗∗daily frequency in number of times tobacco is chewed in a day, ∗∗∗total duration of habit in “years,” ∗∗∗∗for cancer of lip, oral cavity, and pharynx only, n/a: not available, ^+^1–10/day, ^++^>5/day, ^+++^0–19 years, and nonchewers taken as reference category. Frequency/intensity OR are for both genders.

**Table 3:** Epidemiological studies of chewing paan with tobacco and oral cancer.

Authors	OR (95% CI)	Men OR (95% CI)	Women OR (95% CI)	Daily frequency/intensity∗∗			Total duration of use∗∗∗		
				Paan ≤ 5 OR (95% CI)	Paan 6–10 OR (95% CI)	Paan > 10 OR (95% CI)	Paan ≤ 10 yrs OR (95% CI)	Paan 11–20 yrs OR (95% CI)	Paan > 20 yrs OR (95% CI)
Sankaranarayanan et al. [29]^a^	6.1 (3.2–5.7)	3.6∗	6.5∗	3.3 (1.6–6.9)	2.3 (1.2–4.6)	6.1 (2.8–13.2)	4.7∗	2.4∗	5.0∗
Sankaranarayanan et al. [30]^a^	8.7 (3.5–21.4)	9.0∗	11.3∗	4.7 (2.2–10.0)	4.0 (1.9–8.4)	13.2 (6.2–27.8)	3.4∗	4.0∗	14.6∗
Sankaranarayanan et al. [31]^a^	14.1 (7.4–26.5)	10.9∗	7.3∗	6.0∗	9.5∗	15.7∗	7.1 (2.7–18.2)	4.4 (2.4–8.1)	n/a
Wasnik et al. [36]	9.4 (5.1–17.4)	n/a	n/a	n/a	n/a	n/a	n/a	n/a	n/a
Merchant et al. [38]	8.4 (2.3–30.6)	n/a	n/a	n/a	n/a	n/a	n/a	n/a	n/a
Balaram et al. [39]	15.7 (11.0–22.1)	6.1 (3.8–9.7)	45.8 (25.0–84.1)	8.5 (5.4–13.3)	19.4 (10.8–27.0)	24.7 (12.5–48.7)	n/a	n/a	n/a
Subapriya et al. [41]	3.1∗	n/a	n/a	n/a	n/a	n/a	n/a	n/a	n/a
Muwonge et al. [44]	5.4 (3.8–7.7)	3.4 (2.2–5.2)	11.8 (6.0–23.3)	3.7 (2.4–5.5)	5.8 (3.9–8.7)	7.8 (4.8–12.7)	n/a	n/a	5.6∗
Ray et al. [49]	3.9 (2.4–6.4)	1.5 (0.7–3.0)	8.5 (4.6–15.5)	n/a	n/a	n/a	n/a	n/a	n/a

OR: odds ratio, CI: confidence interval, ∗95% CI not reported and/or could not be calculated from given data, ∗∗daily frequency in number of times paan is chewed in a day, ∗∗∗total duration of habit in “years,” n/a: not available, and ^a^the difference between overall and stratum specific OR is because the overall OR and some dose response OR are adjusted for smoking and alcohol while others were calculated using MH method. Nonchewers are taken as reference category. Frequency/intensity OR are for both genders.
