# Supplementary material for: Smokeless Tobacco and Oral Cancer in South Asia: A Systematic Review with Meta-Analysis
Source: J Cancer Epidemiol. 2014 Jul 6;2014:394696. doi: 10.1155/2014/394696 (PMC4109110; doi:10.1155/2014/394696)
Supplement: Supplementary file 1 — The supplementary table: provides additional details about the characteristics of the included publications such as the site of cancer, name of the city/s where the study was conducted, type of controls used (hospital or population), sexes involved in the study and length of the followup for cohort studies etc. [file 394696.f1.pdf]

Supplementary table 1. Additional characteristics of the selected studies.

Study Reference	Author	Type of cancer	Sexes considered	Study location	Type of Controls	Type of exposure	Follow up period
29	Sankaranarayanan et al.	Floor of the mouth and base tongue	Males and females	Kerala	Hospital controls	Smoking, smokeless tobacco and alcohol.	
30	Sankaranarayanan et al.	Labial and buccal mucosa	Males and females	Kerala	Hospital controls	Smoking, smokeless tobacco and alcohol.	
31	Sankaranarayanan et al.	Gingiva	Males and females	Kerala	Hospital controls	Smoking, smokeless tobacco and alcohol.	
32	Goud et al.	Buccal cavity cancer.	Males and females	Varnasi	Two separate hospital based control groups	Different forms of smokeless tobacco.	
33	Nandakumar et al.	Oral cancer	Males and females	Bangalore	Population based controls	Diet, smoking and chewing tobacco.	
34	Rao et al.	Oral cancer	Males	Mumbai	Hospital controls	Alcohol, smoking and smokeless tobacco.	
35	Khan et al.	Oral cancer	Males and females	Karachi	Hospital controls	Lifestyle risk factors	
36	Wasnik et al.	Oral and pharyngeal cancers	Males and females	Nagpur	Hospital controls	Smoking, chewing and occupational exposures.	

**37**	Dikshit et al.	Oropharyngeal , oral and lung cancer	Males	Bhopal	Population based controls	Smoking and smokeless tobacco	
**38**	Merchant et al.	Oral cancer	Males and females	Karachi	Hospital controls	Betel quid with and without tobacco	
**39**	Balaram et al.	Oral cancer	Males and females	Bangalore, Madras, Trivandrum.	Hospital controls	Smoking, Smokeless tobacco, alcohol use and occupational exposures	
**40**	Znaor et al.	Oral, esophageal and pharyngeal cancers	Males	Trivandrum	hospital controls	Smoking, chewing tobacco and alcohol.	
**41**	Subapriya et al.	Oral squamous cell carcinoma	Males and females	Chidambra m	Hospital controls	Various lifestyle risk factors	
**42**	Gangane et al.	Oral cancer	Males and females	Sevagram	Hospital controls	Smoking, smokeless tobacco, diet and alcohol use.	
**43**	Basu et al.	Head and neck cancer	Males and females	Kolkatta	Hospital controls	Smoking and smokeless tobacco	
**44**	Muwonge et al.	Oral cancer	Males and females	Trivandrum	Controls taken from within the Trivandrum cohort	Smoking, smokeless tobacco and alcohol.	

**45**	Jayalekshmi et al.	Oral cancer	Females only	Kerala	N/A	Tobacco chewing	Eight years
**46**	Jayalekshmi et al.	Oral cancer	Males only	Kerala	N/A	Tobacco chewing and bidi smoking	Eight years
**47**	Pednekar et al.	Multiple cancers	Males and females	Mumbai	N/A	Smokeless tobacco	649,228 person years
**48**	Madani et al.	Oral cancer	Males and females	Pune	Hospital controls	Smoking, smokeless tobacco and alcohol.	
**49**	Ray et al.	Oral cancer	Males and females	Kolkata	Hospital controls	Smoking, smokeless tobacco and alcohol.	
